# Preparation and characterization of natural gum nanomuscovite nanocomposite for removal of heavy metals

**DOI:** 10.1038/s41598-026-51409-x

**Published:** 2026-05-29

**Authors:** M. Nageeb Rashed, A. S. A. Arifien, F. A. El-Dowy

**Affiliations:** Chemistry Department, Faculty of Science, Aswan University, Aswan, Egypt

**Keywords:** Arabic gum, Nanocomposite, Adsorption, Lead, Cadmium, Nanomuscovite, Chemistry, Environmental sciences, Materials science, Nanoscience and technology

## Abstract

A common technique to boost the adsorption capability of adsorbent material’s is to alter its composition. In this study, novel nanomuscovite / Arabic gum nanocomposite was synthesized to increase heavy metals adsorption capacity of nanomuscovite. The nanocomposite was fabricated using freeze-drying precipitation by loading gum Arabic (GA) on muscovite nanoparticle. Arabic gum particle was loaded into nanomuscovite with ratios 1:1, 2:1, and 1:2 by weight. The prepared nanocomposite was characterized using transmission electron microscopy (SEM), Fourier transform spectroscopy (FTIR), X-ray diffraction pattern (XRD), scanning electron microscopy (SEM), and BET specific surface area. The effects of various parameters (pH, solution temperature, composite dose, contact time, and metal concentrations) on adsorption capacity of Cd and Pb were studied. Four surface adsorption models were used, including Langmuir, Dubinin-Radushkevich, Temkin, and Freundlich models. The kinetics adsorption of Cd and Pb on the adsorbent surface was investigated using the pseudo first order, pseudo second order, intraparticle diffusion, and Elovich models. It was reported that nanocomposite prepared with a 2:1 w ratio of Arabic gum to nanomuscovite exhibited higher Cd and Pb removal than with the others. Additionally, the maximum Cd and Pb adsorption efficiencies (45.9, 38.01 mgg^-1^, respectively) and high removal percentage (95% and 96%, respectively) were achieved at pH 7, 0.1 g adsorbent dosage, 75 ppm metal concentration, 25 °C solution temperature, and 60 min shaking time. The adsorption of these metals followed the Langmuir model, while the kinetics information fits a pseudo-second order. Thermodynamic calculation of Pb and Cd ion interactions indicate that the reaction is exothermic, as evidenced by the negative free energy ΔG° value, which suggests that the process occurs spontaneously.

## Introduction

In the past few years, growing concerns have emerged regarding environmental pollution and public health problems linked to both organic and inorganic contaminants. Because of the harmful effects of heavy metal ions on human health and ecosystems, there has been significant interest in their removal from wastewater^1,2^.

Water pollution poses a worldwide issue as various elements have led to deterioration in water quality. Contributing factors include swift urban growth and industrial development, a rising global population, and the reckless use of natural resources^3,4^. Heavy metal ions, known for their toxicity and carcinogenic properties, are the prevalent non-biodegradable pollutants that build up in living organisms. Cd and Pb are two examples of toxic heavy metals that are non-biodegradable^1,5^.

There are various methods for heavy metal removal from polluted water. Some of these techniques include activated carbon adsorption, solvent extraction, chemical electrode, ion-exchange, and precipitation, followed by adsorption. Adsorption has garnered the most interest due to its simplicity, low cost, and effectiveness, particularly in treating wastewater^6^.

An appropriate low-cost adsorbent must possess a porous framework, along with mechanical and chemical durability, and a strong attraction to the contaminants of interest^7^. Natural ore, clays, and rocks were utilization as non-conventional adsorbents^8,9^.

Natural polymers have been utilized in synthesis of nanocomposites because of their beneficial characteristics, including being non-toxic, biodegradable, biocompatible, and others^10,11^. Furthermore, polymers derived from natural sources are renewable, unlike those from petrochemical origins, which are not easily broken down by the environment^12^.

Natural polymers are utilized in pollutant removal from water because they are cost-effective, biodegradable, biocompatible, and possess a high adsorption capacity^13^. One method involves the development of adsorbents using natural polymers that are modified with monomers and nanoparticles, resulting in the formation of additional active sites that interact with specific pollutants, which enhances the surface area^14^.

Arabic Gum is a natural substance that contains a mixture of inorganic salts and polysaccharides. The inorganic salts includes calcium, potassium and, magnesium while the organic part include β-d-galactopyranosyl 1.3 repeat unit, while the side chains are 2 to 5 units of β-d-galactopyranosyl 1.3 attached to the main chain by 1.6 links^15^. The presence of carboxyl groups in the gum may enhance its ability to adsorb substances, making it an effective natural adsorbent^16^.

Arabic gum is a widely recognized natural substance with many different applications. It finds extensive use in the food, cosmetic, and pharmaceutical sectors. Additionally, it has applications as stabilizer and emulsifier. In certain developing nations, Arabic Gum is employed to address chronic kidney disease^17^.

Several studies reported that Arabic gum can be utilized for the synthesis of different nanocomposite materials that are technologically significant, owing to its non-toxic properties, high solubility, low viscosity, and excellent emulsifying qualities^18^. Elwakeel et al.^3^ prepared hydrogel beads by casting a mixture of alginate and Arabic gum (in the absence and presence of a nanomagnetic core) and applied it as adsorbents to remove Cd, and Pb from water. Ali et al.^19^ prepared gum Arabic-magnetite nanocomposite as adsorbent for Pb removal from polluted water. Sahraei and Ghaemy^20^ prepared Gum Tragacanth loaded Graphene Oxide composite for the treatment of wastewater from heavy metals. Abreu et al.^21^. prepared core-shell chitosan-Arabic gum NPs, and applied it for Cu removal from solution. Gum Arabic-mushroom composite has been prepared as biocomposite and applied it as biosorbent for treatment metals (Ag, Zn) from wastewater^22^.

This study aims to prepare a novel nanomuscovite / Arabic gum nanocomposite using nomuscovite for the first time, in which no research published about nanomuscovite / Arabic gum nanocomposite. The prepared nanocomposite will undergo the adsorption experiments for the removal of Cd and Pb from polluted water, and to adjust the optimum conditions for the metals maximum adsorption. In this study, the modification aims to enhance the electrostatic properties and surface area of the nanomuscovite by incorporating natural gum onto the surface of nanomuscovite.

## Materials and methods

### Materials

Muscovite was collected from the Hafafit region, located in the southern part of the eastern desert of Egypt. Five bulk samples, each weighing 5 kg, were taken from this location. The samples were crushed and milled into a powder that reached a 65 μm particle size, and then dried at 60 °C for 24 h. The elemental composition of muscovite shown in Table 1. Arabic gum was sourced from the acacia tree.

**Table 1:** Chemical composition of raw Muscovite sample by XRF.

SiO_2_%	Na_2_O%	K_2_O%	Al_2_O_3_%	Cd ppm	Zn ppm	Pb ppm	Cu ppm
48.16	0.72	10.62	32.5	12.70	0.482	0.02	0.04

**Table 2:** Ratio of Arabic gum and nano-muscovite.

Gum Arabic / nanomuscovite ratio	Arabic gum solution ( ml)*	Nano-muscovite (g)
1:1 [GA/Msc1]	20	1.8
2:1 [GA/Msc2]	20	0.9
1:2 [GA/Msc3]	20	3.6

*solution of 9 gm Arabic Gum in 100 deionized water, 20 ml contains 1.8 g Arabic Gum.

All chemicals used were of high analytical grade. Alfa Aesar diethylene triamine penta-acetic acid DTPA (C1_4_H_23_N_3_O_10_). lead nitrate Pb(NO_3_)_2_, and cadmium nitrate Cd(NO_3_)_2_ were obtained from Sigma-Aldrich.

### Synthesis of Pb^2+^ and Cd^2+^ standard solutions

Working standard Pb^2+^ and Cd^2+^ solutions were prepared through diluting 1000 ppm stock one.

### Instruments

Pb^2+^ and Cd^2+^ concentrations were determined by atomic absorption spectrophotometer (GBC 932AA). Instruments for materials characterization are EDXRF (JOEL JSX 3222), XRD ( Brukeraxs D8, Germany), SEM-EDX ( JEOL, JSM-5500 LV electron microscopy), FTIR (Cary 630 FTIR spectrometer, Agilent technologies Company, spectral range wavenumbers from 4000 cm^− 1^ to 400 cm^− 1^ ), and TEM (TEM, JEOL-JEM-1230, Tokyo, Japan).

### Preparation of activated of muscovite

The preparation of activated Muscovite was processed as in the published paper by Rashed et al.^23^. To 200 mL HCl (0.8 M), 5 g of purified muscovite was added. The mixture was stirred for 10 h at 25 °C, filtered, washed with DW, and dried at 100 °C. The resulted precipitate was mixed with 30% H_2_O_2,_ with stirring for 7 h. After filtration and, washed with deionized water (DW) it was dried for 10 h at 100 °C, and ground to the size 63 μm.

### Nanomuscovite preparation

According to our published method^23^ nanomuscovite was prepared. Activated muscovite (5 g) and 6.475 g of DTPA was mixed with100 mL DW and stirred at 70 °C for 3 days. After filtration the precipitate was washed with 0.1 M NaOH until pH 7, followed by DW and dried for 10 h at 100 °C.

### Preparation of Arabic gum Nano-muscovite nanocomposite

Gum Arabic stock solution was prepared by dissolving 9 g Gum powder in 100 ml DW and stirred at 50 °C for 2 h. A different amount of Nano-Muscovite was added to the prepared gum solution in the ratio 1:1, 2:1 and 1:2 according to Table (2), and label as 1:1 GA/Msc1, 2:1 GA/Msc2, and 1:2 GA/Msc3, respectively .

After then it was stirred for 3 h at 500 rpm. Freeze-drying technique was applied, in which the homogeneous mixture cast into a 5 ml plastic cylinder (12–40 mm), frozen for 24 h in 20 °C, followed by 24 h at 100 °C^24^.

### Comparison study of the Arabic gum nanocomposite for removal of the metals

Adsorption of Cd^2+^ and Pb^2+^ on the various prepared Arabic gum nanocomposite (GA/Msc1, GA/Msc2, GA/Msc3) was studied at the optimum conditions to compare the different nanocomposites for its best metal removal. The metal removal (R%) and equilibrium qe (mg g^− 1^) obtained by these equations 

1 $${\mathrm{R}}\% {\text{ }}=\left( {{{\mathrm{C}}_{\mathrm{o}}} - {{\mathrm{C}}_{\mathrm{e}}}} \right){\text{ }}/{{\mathrm{C}}_{\mathrm{o}}}\,{\mathrm{X}}\,{\mathrm{1}}00$$

2 $${{\mathrm{q}}_{\mathrm{e}}}=\left( {{{\mathrm{C}}_{\mathrm{o}}} - {{\mathrm{C}}_{\mathrm{e}}}} \right){\mathrm{V}}/{\mathrm{m}}$$

Where C_e_ ,C_o_ ,V and m are equilibrium metal concentrations (ppm), initial metal concentration (ppm), metal solution volume (L) and adsorbent dosage (g), respectively.

All the experiments were performed in triplicates, and the average values were used for experimental data evaluation.

### Optimum conditions for the maximum adsorption of the metals on Arabic gum nanocomposite (GA/Msc2)

#### pH effect

Adsorption experiments to the impact of solution pH were carried out as described below. The influence of pH were on removal of Cd^2+^ and Pb^2+^ was investigate following this experiment : 0.1 g of (GA/Msc2) nano-composite was stirred with 50 ml of 75 ppm of Cd^2+^ and Pb^2+^ for 2 h, at different pH 2, 4, 5, 6, 7, 8 and 10. After filtration Cd^2+^ and Pb^2+^ were measured in the filtrate by AAS.

#### Adsorbent dosage effect

Different amounts (0.05, 0.1, 0.2, 0.3, 0.5 and 1.0 g) of GA/Msc2 nanocomposite was stirred with 50 ml of 75 ppm of Cd^2+^ and Pb^2^ for 2 h, and adjusted at pH 6 and 7 for Pb^2+^ and Cd^2+^, respectively. After filtration the metal were measured by AAS.

#### Initial metal concentration effect

0.1 g of GA/Msc2 nanocomposite was stirred with 50 ml of different Cd^2+^ and Pb^2+^ concentration (10, 20, 30, 50, 75 and 100 ppm) at 25 °C for 2 h at pH 6 and 7 for Pb^2+^ and Cd^2+^, respectively. After filtration, the metal were measured by AAS.

#### Contact time on adsorption effect

For study the time effect on metal adsorption, 0.1 g GA/Msc2 nanocomposite was stirred with 50 ml of 75 ppm of Cd^2+^ and Pb^2+^ at optimum conditions (pH 6 and 7 for Pb^2+^ and Cd^2+^, respectively, and solution temperature 25 °C) for 30, 60, 120 and 180 min. After filtration, the metal was measured by AAS.

#### Temperature effect

The influence of the metal solution temperature on adsorption was studied in which 0.1 g of GA/Msc2 nanocomposite was stirred with 50 ml of 75 ppm of Cd^2+^ and Pb^2+^ at optimum conditions (pH 6 and 7 for Pb^2+^, and Cd^2+^, respectively, and contact time 2 h ) at temperature range 25–55 °C. After filtration, the metal ions were measured by AAS.

#### Adsorption isotherms, kinetics and thermodynamic models

Grasping interaction between adsorbents and pollutants is crucial for adsorption research. Adsorption isotherms models (Freundlich, Langmuir, Dubinin-Radushkevich and Temkin) were examined. Among these models, the Freundlich and Langmuir isotherms are frequently utilized to determine Q_o_ and qe .

These models are :

3 $${\text{Langmuir model }}{{\mathrm{C}}_{\mathrm{e}}}/{{\mathrm{q}}_{\mathrm{e}}}={\text{ 1}}/{{\mathrm{Q}}_{\mathrm{o}}}{\text{b }}+{\text{ }}{{\mathrm{C}}_{\mathrm{e}}}/{{\mathrm{Q}}_{\mathrm{o}}}$$

where Ce, and qe are metal concentration and equilibrium adsorption capacity, respectively, Q_o_ maximum adsorption capacity, b Langmuir constant

4 $${\text{Freundlich model Log }}{{\mathrm{q}}_{\mathrm{e}}}={\text{ log }}{{\mathrm{K}}_{\mathrm{f}}}+{\text{ 1}}/{\text{n log }}{{\mathrm{C}}_{\mathrm{e}}}$$

K_f,_ Freundlich isotherm constant the adsorption capacity and n the adsorption intensity

5 $${\text{Temkin model }}{{\mathrm{q}}_{\mathrm{e}}}={\text{ B ln K}}\,+\,{\text{B ln }}{{\mathrm{C}}_{\mathrm{e}}}$$

B Temkin constant related to heat of sorption., and q_e_ (mg/g) the amount of adsorbed heavy metals per unite weight of adsorbent

6 $${\mathrm{Dubinin}} - {\mathrm{Radushkevich}}{\mkern 1mu} \,{\mathrm{model}}{\mkern 1mu} {\mathrm{(D}} - {\mathrm{R)Lnq}}_{{\mathrm{e}}} = {\text{ln q}}_{{\mathrm{m}}} - {\mathrm{BE}}^{{\mathrm{2}}}$$

7 $${\mathrm{E}}\,=\,{\text{RT ln }}({\mathrm{1}}\,+\,{\mathrm{1}}/{\mathrm{Ce}})$$

8 $${\mathrm{E}}\,=\,{\mathrm{1}}/{\text{ }}\left( {{\mathrm{2B}}} \right)^{{\raise0.5ex\hbox{$\scriptstyle 1$}\kern-0.1em/\kern-0.15em\lower0.25ex\hbox{$\scriptstyle 2$}}}$$

R, constant ideal gas (8.314 J/mol K), T absolute temperature (K), q_m_ constant of the adsorption degree (mg/g), B constant adsorption energy (mol/kJ), and E the polanyi potential.

### Adsorption kinetic models

For modeling the kinetics of Cd^2+^ and Pb^2+^ adsorption, and to find the rate of adsorption process, various models were applied include pseudo-first order, pseudo-second order, intraparticle diffusion, and Elovich models.

#### Pseudo–first order

9 $${\mathrm{Log~}}\left( {{\mathrm{q~}}_{{\mathrm{e}}} {-}{\mathrm{q}}_{{\mathrm{t}}} } \right) = {\mathrm{log~q}}_{{\mathrm{e}}} {-}{\mathrm{~}}\left( {{\mathrm{K}}_{1} /2.303} \right){\mathrm{~t}}$$

#### Pseudo–second order

10 $${\mathrm{t}}/{\mathrm{q}}_{{\mathrm{t}}} = {\text{ 1}}/{\mathrm{K}}_{{\mathrm{2}}} {\mathrm{q}}_{{\mathrm{e}}} ^{{\mathrm{2}}} + {\mathrm{1}}/{\mathrm{q}}_{{\mathrm{e}}} {\mathrm{t}}$$

#### Intra-particle diffusion

11 $${\mathrm{q}}_{t} = {\mathrm{K}}_{i} {\text{t~\raise.5ex\hbox{$\scriptstyle 1$}\kern-.1em/ \kern-.15em\lower.25ex\hbox{$\scriptstyle 2$} ~}} + {\mathrm{~C}}$$

#### Elovich

12 $${\mathrm{q}}_{{\mathrm{t}}} = 1/\beta {\mathrm{~ln}}\left[ {\alpha \beta } \right] + {\mathrm{~}}1/\beta {\mathrm{~ln~t}}$$

where K1 rate constant, the adsorption capacities qe (mgg^-1^), adsorption equilibrium qt (mgg^-1^) and equilibrium time t (min). The rate constant K2 (gmg^-1^min^-1^), C (mgg^−1^) the boundary layer effect and thickness, Ki the intra-particle diffusion rate constant (mg g^−1^ min^−1/2^). α the initial adsorption rate, and β the adsorption coefficient

#### Thermodynamics

The thermodynamics of Cd^2+^ and Pb^2+^ adsorption on the prepared GA/Msc2 nanocomposite was carried out at range temperatures 298 - 333 K. Free energy ΔG◦, enthalpy ΔH◦, and entropy ΔS◦ were obtained using Eqs.13 and 14^25^

13 $${\mathrm{Ln}}\,{\mathrm{K}}\,{\text{ = }}\,\Delta {\mathrm{S}}^{ \circ } \,{\mathrm{/R}}\,{-}\Delta {\mathrm{H}}^{ \circ } /{\mathrm{RT}}$$

The free energy ∆Go (KJ mol^− 1^) was calculated by this equation

14 $$\Delta {\mathrm{G}}^{ \circ } \, = \Delta {\mathrm{H}}^{ \circ } - {\mathrm{T}}\Delta {\mathrm{S}}^{ \circ }$$

Where K is the adsorption equilibrium constant , R is the ideal gas constant [8.314 J/(mol. K)], and T is the absolute temperature (K). ∆Go (kJ/mol.), ∆So (J/(mol. K)), and ∆Ho (kJ/mol.) are the change in Gibbs free energy, entropy, and enthalpy, respectively

## Results and discussion

### Comparison of GA/Msc nanocomposites for Cd^2+^ and Pb^2+^removal efficiency

For comparison the adsorption efficiency of Pb^2+^ and Cd^2+^ between the prepared GA/Msc nanocomposites (GA/Msc1, GA/Msc2 and GA/Msc3) it is clear that GA/Msc2 nanocomposite exhibited higher removal of Pb^2+^ and Cd^2+^ (76.9% and 74.3%, respectively) than with GA/Msc1, and GA/Msc3 ( Table 3). So it is indicate that Gum Arabic/ nanomuscovite ratio 2:1 (GA/Msc2) is the best for preparing Gum Arabic/ nanomuscovite composite, so it will be used for the further experiments.

**Table 3:** Removal percentage of Pb^2+^ and Cd^2+^ by Gum Arabic muscovite (GA/Msc) nanocomposites adsorbents.

Gum Arabic/ nanomuscovite ratio	% Pb removal	% Cd removal
1:1 [GA/Msc1]	69.7%	66.8%
2:1 [GA/Msc2]	71.9%	69.3%
1:2 [GA/Msc3]	65.7%	63.7%

### Characterization of GA/Msc2 nanocomposite

After selecting GA/Msc2 nanocomposite for the further experiments, it was characterized by several techniques include TEM, FTIR, XRD, SEM, and BET specific surface area .

#### XRD analysis

The modification procedures could be proved by investigating XRD patterns of the nanomuscovite and GA/Msc nanocomposite powders before being formulated than comparing with the patterns of GA/Msc2 nanocomposite.

**Fig. 1:**
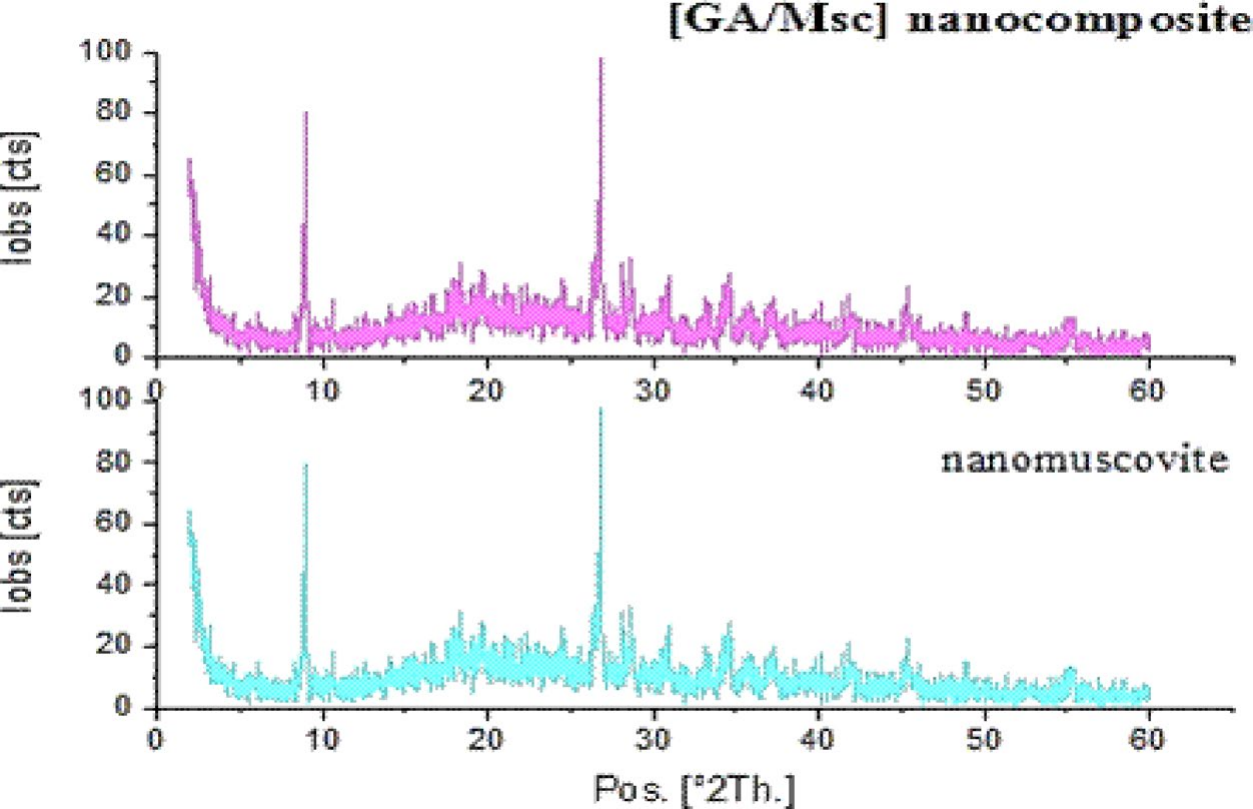
XRD of nanomuscovite and GA/Msc nanocomposite.

XRD of GA/Msc2 nanocomposite was analysed in the 2Ө range of 0° to 60° by the JCPDS instrument (JCPDS: 00-058-2016). The X-ray diffraction patterns for GA/Msc2 nanocomposite and nanomuscovite are shown in Fig. 1.

The XRD graph of nanocomposite shows two peaks at 2θ ( 8.9˚ and 26.8˚), less intense one at 2θ (17.8˚, 26.6˚, 35.9˚) and at 2θ 45.3˚^23^. The XRD graph of GA/Msc2 nanocomposite revealed the same characteristic peaks as nanomuscovite but with low 2θ value. The shift of the diffraction peaks to lower values of 2θ indicates the successful embedding of the Arabic gum chains in the GA/Msc nanocomposite. This effect was similarly noted by Wangmene et al.^26^.

The size of GA/Msc2 nanocomposite crystallite calculated using Scherrer’s formula, and it was in the range of 14.2–52 nm.

The mean crystallite size (D) of the particles was calculated from XRD line broadening measurement using the Scherrer equation.

15 $${\mathrm{D}}\,=\,0.{\mathrm{89}}\,\lambda /\beta \,{\mathrm{cos}}\,\theta$$

D is grain size (nanometers), K is constant, λ is X-ray wavelength, β is the width of the peak at half peak height (phase peak) in radians and θ is diffraction angle in degrees.

#### FTIR

**Fig. 2:**
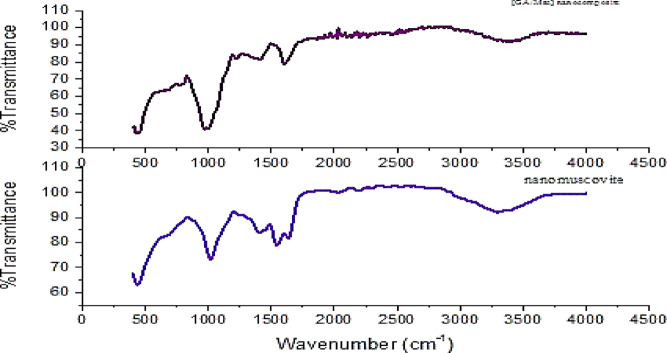
FTIR of nanomuscovite and GA/Msc2 nanocomposite.

FTIR spectra of nanomuscovite and nanomuscovite loaded Arabic gum [GA/Msc2], recorded in the range of 400–4000 cm^− 1^, are presented in Figure 2. For nanomuscovite and [GA/Msc2] nanocomposite spectrum display a peak of Si-O bond stretching at ~ 1016 cm^− 1^. The two bands at 2922 and 2855 cm^− 1^ are associated with the CH_2_ asymmetric vibrations. The modes symmetric stretching vibration of the alkyl chain from the quaternary ammonium modifier is observed, along with another peak corresponds to the bending of the Mg-O bond at 440 cm^− 1^. The bending vibrations associated with the Al-O bond can be seen at 918 cm^− 1^. The peaks found at 1635 and 3298 cm^− 1^ are linked to the vibrations of the hydration water (HOH) and the -OH groups that are present between octahedral sheets and tetrahedral^27^. Peak around 793 cm^− 1^ is associated with bending vibrations of the OH groups linked to the silicate layer in the nanomuscovite structure^28^. Main characteristic bands of Arabic gum C–O stretch are at 1030 and 1429 cm^− 1^, C–O stretch and N–H bending at 1631 cm^− 1^, C–H stretch at 2924 cm^− 1^, and broad absorption band between 3000 and 3600 cm^− 1^ is assigned to peak of –OH groups stretch of the polysaccharide^29^. The weaker peak located at 2130 cm^− 1^ is attributed to the C–O stretching of different carbonyl compounds present in the gum observed in the composite.

#### SEM analysis

**Fig. 3:**
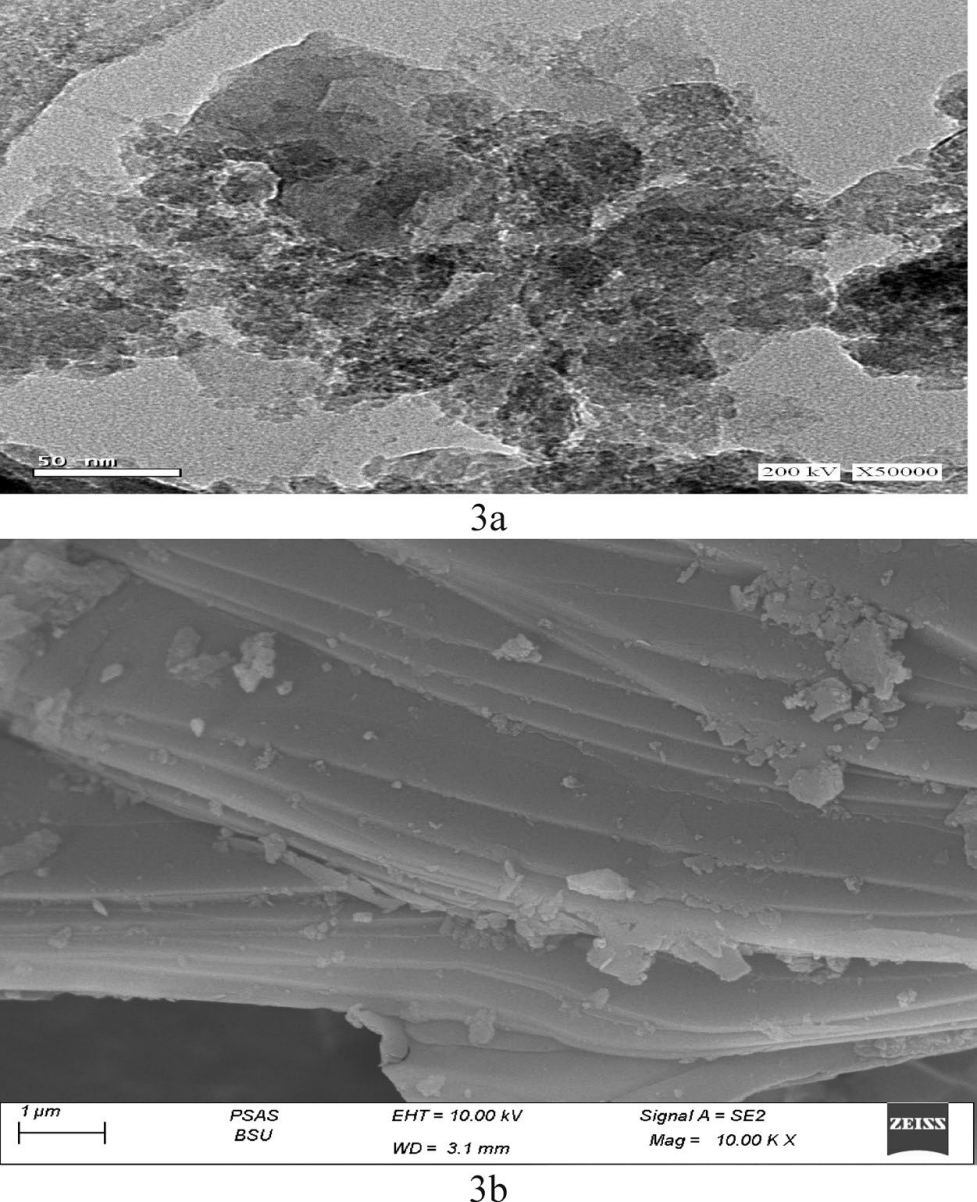
Images SEM of nanomuscovite (**a**) and GA/Msc2 nanocomposite (**b**).

The shape of the resulting GA/Msc2 nanocomposite was characterized and morphological investigations using the SEM technique (Fig. 3). The SEM image of the nanocomposite revealed layered structure of the particles (Fig. 3a). Micrographs of GA/Msc2 (Fig. 3b) illustrate a uniform distribution of GA/Msc2 throughout the composite, exhibiting a smaller particle size than the GA particles. Additionally, the findings revealed notable changes in the morphology of the particle surfaces, characterized by distinct surface pores and a consistent distribution of Msc2 within the GA/Msc2, which may enhance the adsorption of metal ions synergistically. The results presented in Fig. 3b clearly indicate that the polymerization of GA primarily occurred between the layers of the Msc2 nanocomposite. This finding shows that Msc2 readily integrated between the layers of Arabic gum, leading to expansion and the separation of the layers, resulting in the formation of intercalated layered silicate Msc2 particles.

#### BET surface area and BJH

The resulting BET curves yielded important data, summarizing surface area, average pore diameter, and pore volume. This detailed information regarding nanomuscovite and GA/Msc2 nanocomposite is presented in Table 4. The resulted data reveals that the surface area value for nanomuscovite was 195.15 m^2^/g, while that for GA/Msc2 was 203m^2^/g, which reveals the increase GA/Msc nanocomposite surface area than the nanomuscovite.

**Table 4:** The pore size, pore volume, and surface area of prepared GA/Msc2 nanocomposite sample.

Sample	Surface area (m^2^/g)	Pore size (nm)	Pore volume (cm^3^/g)
nanomuscovite	195.15	13.82	0.21
Gum Arabic muscovite nanocomposite (GA/Msc2)	203	13.78	0.23

The pore size of the GA/Msc2 was slightly smaller (13.78 nm) than that of the nanomuscovite (13.82 nm) as a result of particle size reduction. The loading of Arabic gum GA and the nanomuscovite (i) decreases the pore size of the network and swelling index^30^, and (ii) both nanomuscovite and Arabic gum has a synergistic effect in metal adsorption^31^.

### Optimum conditions for Pb^2+^ and Cd^2+^ adsorption on GA/Msc2 nanocomposite

#### pH Influence

pH of the solution has vital effect in metal adsorption process. It significantly influences the metal adsorption due to the surface charge of the adsorbent, speciation of adsorbate during, and degree of ionization^8,32^.

**Fig. 4:**
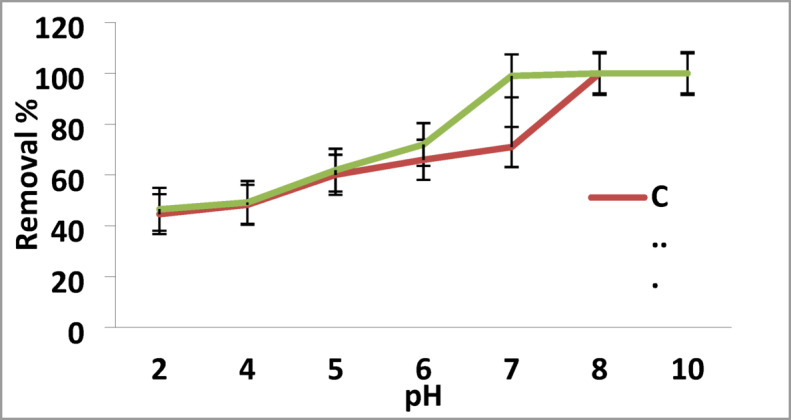
Removal of Cd^2+^ and Pb^2+^on GA/Msc2 nanocomposite according to pH.

The influence of pH on Pb^2+^ and Cd^2+^ adsorption by the newly designed GA/Msc2 nanocomposite was proceed with pH range 2.0 to 10.0 at 25 °C (Fig. 4). The maximum removal percentage of Pb^2+^ and Cd^2+^ was found at pH 6.0(72%) and at pH 7.0 (71%) for Pb^2+^ and Cd^2+^, respectively. At lower pH levels, the reduction in metal adsorption is mainly due to a higher concentration of H^+^ ions in the solution, which compete with metal ions for available adsorption sites on the adsorbent. In contrast, at elevated pH levels, the decrease in adsorption results from the creation of soluble hydroxyl complexes^33^.

The pHPZC of GA/Msc2 nanocomposite was found to be 3.5. At pHPZC, the surface of the nanocomposite is negatively charged that enhances the adsorption of Pb onto the surface of the nocomposite.

#### Influence of adsorbent dose

**Fig. 5:**
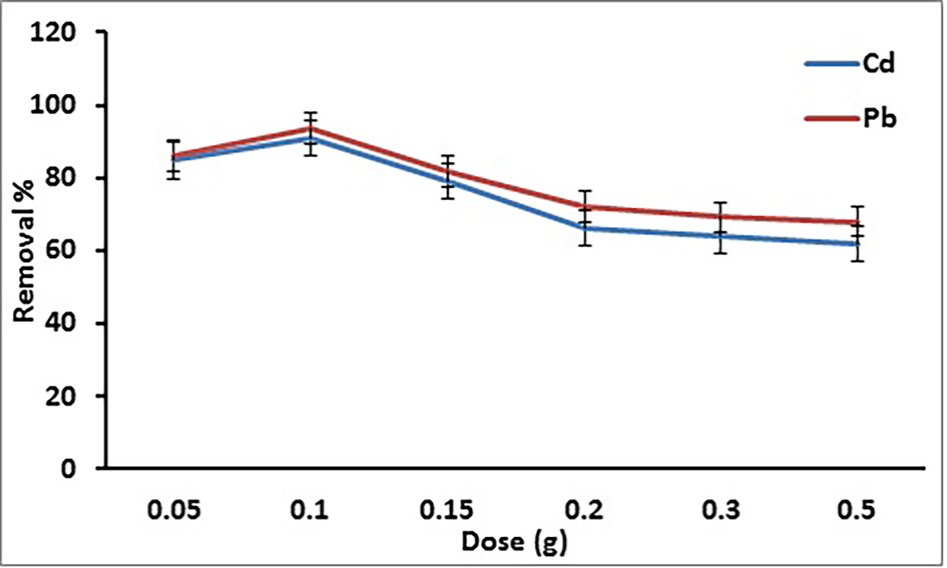
Removal of Cd^2+^ and Pb^2+^on on GA/Msc2 nanocomposite according to dosage.

The removal of Cd^2+^ and Pb^2+^ ions versus the GA/Msc2 nanocomposite dosage (0.05–0.5 g) is demonstrated at Fig. 5. The figure shows that the Cd^2+^ and Pb^2+^ ions removal sharply rises with increasing adsorbent amount from 0.05 to 0.1 g and reaching maximum for Cd^2+^ and Pb^2+^ ions at 0.1 g GA/Msc2 (91% and 93%, respectively). After then the metal removal decrease. So, the optimum GA/Msc2 nanocomposite dosage for the removal of Cd^2+^ and Pb^2+^ ions was 0.1 g.

The increase in metal removal may be linked to that as the dosage of adsorbent increases, the number of adsorption sites available also raises, which improves the elimination of metal ions. The reduction in the adsorption of metal with increasing adsorbent dose could related to the overlapping and clustering of adsorption sites caused by adsorbent particles, resulting in a decrease in the overall surface area at higher doses. Additionally, it might be due to the weaker binding of the adsorbate to the adsorbent’s surface^34^.

#### Influence of initial metals concentration

**Fig. 6:**
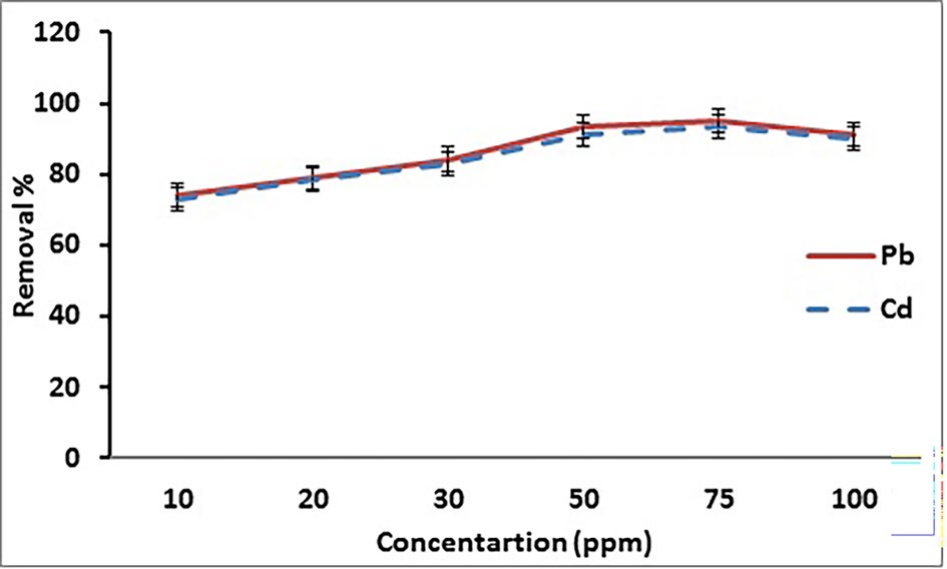
Removal of Cd^2+^ and Pb^2+^on GA/Msc2 nanocomposite according to initial concentration.

The percentage adsorption efficiency of Cd^2+^ and Pb^2+^ as function of initial concentration is shown in Fig. 6. The result reveals that the highest removal efficiency of Cd^2+^ and Pb^2+^ was 93.5% and 94.9% at 75 mg /L for Cd^2+^ and Pb^2+^, respectively.

Thus, the diffusion of metal ion towards the adsorbent sites is important and therefore retention becomes more necessary. Increasing the concentration of metal ion may enhance a specific concentration gradient force, which can lead to accelerated molecular diffusion towards the surface of the adsorbent. Therefore, as the concentration of the metal ion increases, a greater number of these metals can reach the active sites of the adsorbent, leading to an increase in adsorption levels^35^.

#### Influence of contact time

**Fig. 7:**
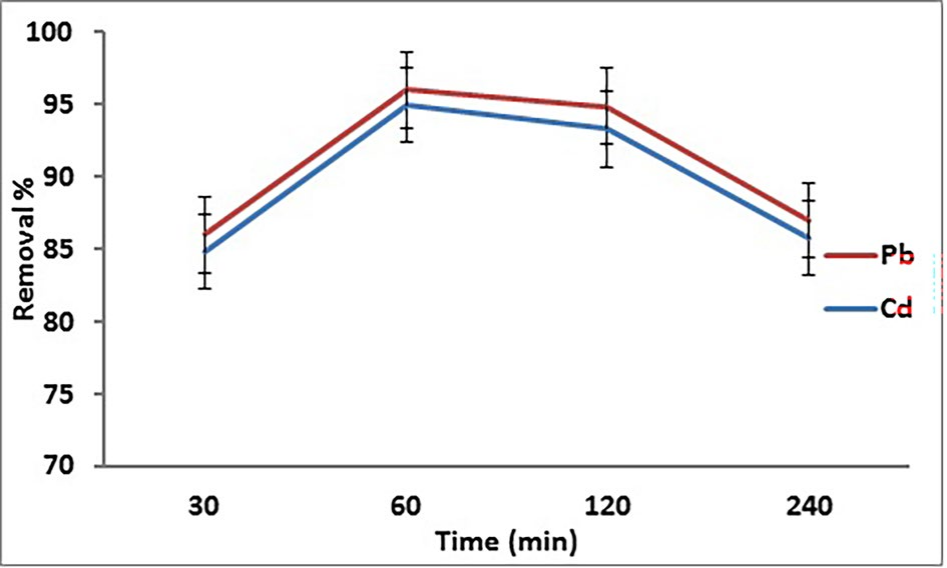
Removal of Cd^2+^ and Pb^2+^on GA/Msc2 nanocomposite according to contact time.

The contact time dependency of the removal procedure of Cd^2+^ and Pb^2+^ by GA/Msc2 is given in Fig. 7. Figure 7 shows that the removal of Cd^2+^ and Pb^2+^ ions by the prepared GA/Msc2 increased with contact time. A rapid incorporation of Cd^2+^ and Pb^2+^ ions occurred by GA/Msc2 in 15 min, indicating high affinity between the metal ions and the adsorbent. Also, metals adsorption capacity gradually increased with time, and after 60 min a maximum adsorption of Cd^2+^ and Pb^2+^ (84.8% and 86%, respectively) was observed. This is as result of the repulsion taking place on the surface of the GA/Msc2 adsorbent, caused by the occupation of the adsorption sites by the metal ions^36^.

#### Temperature influence

The influence of solution temperature on sorption of Cd^2+^ and Pb^2+^ by GA/Msc2 nanocomposite was examined by varying the temperature from 25 to 55 °C at constant optimum conditions (pH 6–7, 0.1 g adsorbent dose, 60 min contact time, and 75 mg L^− 1^ metal ion concentration ). Results ( Table 5) show that the percentage adsorption efficiency of Cd^2+^ and Pb^2+^ decreased slowly as temperature increased. The highest removal of Cd and Pb (95% and 96%, respectively) was at 25 °C. The reduction in metal ions adsorption efficiency with rising temperature could also attribute from block of some active sites of the adsorbents^37^.

**Table 5:** Removal of Cd^2+^ and Pb^2+^on GA/Msc2 nanocomposite according to temperature.

Temp. °C	Removal % Pb	Removal % Cd
25	96	95
35	88.6	88
45	76.2	75
55	67.2	66

The reduced efficiency of metal adsorption at higher temperatures may result from the destabilization of the interaction between the physical adsorbate and adsorbent, prompted by the increased energy of the medium. This destabilization could lead to a partial binding of the metal ions onto the adsorbents^38^.

In conclusion the optimum conditions for the maximum adsorption of Cd^2+^ and Pb^2+^ (95% and 96%, respectively) was at pH 7, 0.1 g adsorbent dosage, 75 ppm metal concentration, 25 °C temperature, and 60 min shaking time. Comparing this results of GA/Msc2 nanocomposite with its one component (nanomuscovite), it reveal that the removal of Cd^2+^ and Pb^2+^ on GA/Msc2 nanocomposite was higher (95% and 96%, respectively) than that with the nanomuscovite (91.5% and 95%, respectively )^23^.

### Adsorption isotherms

The interaction between metal ions and the adsorption sites of the GA/Msc2 nanocomposite can be analysed through adsorption isotherm models^19^. These isotherms are defined by specific factors which indicate the surface characteristics and the affinity of the adsorbent for metal ion adsorption.

The obtained data from isotherm models (Freundlich, Langmuir, Dubinin-Raduskevich D-R, and Temkine) at optimum conditions (0.1 g adsorbent dosage, solution temperature 25 °C, 60 min contact time, and initial metals concentration 75 ppm) are presented in Table 6.

**Table 6:** Results of adsorption isotherm plot parameters for adsorption of Cd^2+^ and Pb^2+^on GA/Msc2 nanocomposite.

Parameters	Cd^2+^	Pb^2^
Langmuir Isotherm model		
Q_0_ (mg/g)	38.01	45.9
b_L_ (L/mg)	0.05	0.21
R^2^	0.98	0.97
Freundlich Isotherm model		
n	2.17	1.78
K_f_ (mg^− 1/n^ L^1/n^ g^− 1^)	2.5	2.6
R^2^	0.77	0.805
Temkin Isotherm model		
B (j/mol)	11.1	10.4
b_T_(j/mol)	226	241
Kt (L/g)	1.26	1.55
R^2^	0.95	0.95
Dubin-Raduskevich Isotherm model		
q_D_ (mg/g)	51.9	19.3
E (kj/mol)	0.5	0.4
R^2^	0.83	0.86
β (mol/kJ)	2*10^− 6^	4*10^− 6^

#### Langmuir isotherm

**Fig. 8:**
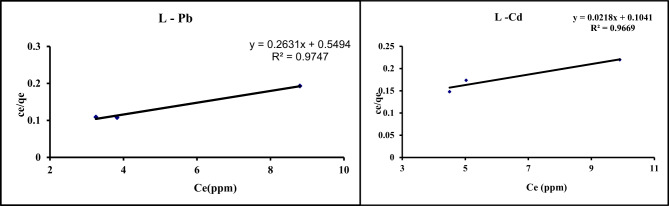
Langmuir isotherm for the adsorption of Pb and Cd on GA/Msc2 nanocomposite.

From Eq. (1), by plotting Ce/qe vs. Ce, the Q_max_ and b values are obtained from the slope and intercept (Fig. 8), and tableted in Table 6. Results of R^2^for Pb and Cd (0.970 and 0.980, respectively) were high indicted that the adsorption of the metals obeyed the Langmuir model. The obtained qmax values for Pb and Cd were 45.9 and 38.01 mg/g, respectively, which was very close to the values found at the optimal pH value. The obtained b value (0.21 L/mg for Pb and 0.05 L/mg) revealed a high attraction between the GA/Msc2 nanocomposite and Pb or Cd ions with more binding affinity between the GA/Msc2 nanocomposite and the metals^18^.

This model illustrates the adsorption of a solitary layer onto the absorbent surface, presuming that there is uniform and equal interaction between all adsorbent sites and pollutants, resulting in homogeneous adsorption^39^.

#### Freundlich isotherm

**Fig. 9:**
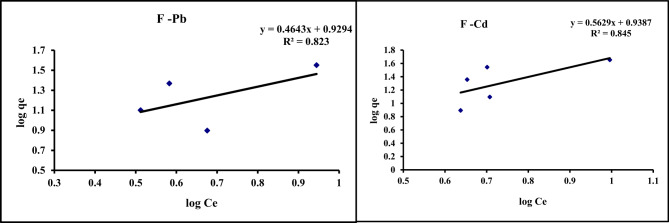
Freundlish isotherm for the adsorption of Pb and Cd on GA/Msc2 nanocomposite.

From Eq. 2, by plotting log q_e_ vs. log C_e_ (Figure 9), K_f_ and n were are obtained from the slope and intercept, and tableted in Table 6. The data represented that the value for *n* < 1 in Freundlich isotherm for Cd and Pb were 2.17 and 1.78, respectively. This prove favourable physical adsorption of Cd and Pb on GA/Msc2 nanocomposite^39^**.**

This isotherm model describes the reversible adsorption process on adsorbent surfaces that have differing characteristics. It views the adsorbent surface as heterogeneous, featuring active sites that have distinct energy levels.

#### Temkin isotherm

**Fig. 10:**
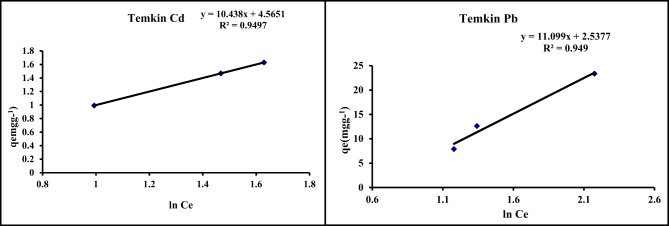
Temkin isotherm for the adsorption of Pb and Cd on GA/Msc2 nanocomposite.

This isotherm model forecasts a consistent spread of binding energies throughout the surface binding adsorption population. From Eq. (3), by plotting q_e_ vs. ln C_e_ (Fig. 10; Table 6) B (constant related to the heat of sorption J/mol) and Kt (binding constant ) were obtained. The binding constant (Kt) values for Pb (1.55) and Cd (1.26) indicate that Pb binds more strongly than Cd, which is attributed to its larger hydrated ionic radii and higher electronegativity in solutions of metallic species^40^.

#### Dubinin-Radushkevich isotherm model

**Fig. 11:**
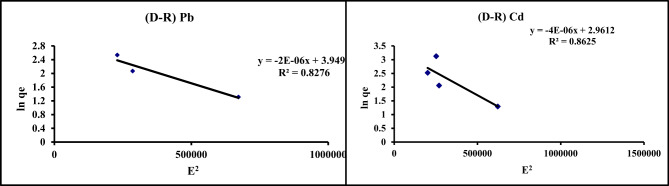
D-R isotherm for the adsorption of Pb and Cd on GA/Msc2 nanocomposite.

From Eq. (4) β and q_m_ were obtained by plotting ln qe vs. E^2^ (Fig. 11). The results from Table 6 reveal that E values for Pb and Cd adsorption lies under 8 and 16 kJ mol ^− 1^ prove that the adsorption is reversible, and the saturation of GA/Msc2 sites by the Pb and Cd ions^6^. This isotherm model is typically used to describe the adsorption process characterized by a Gaussian energy.

In conclusion, from Table 6, the adsorption of Cd^2+^and Pb^2+^ on GA/Msc2 nanocomposite fitted Langmiur Isotherm due to R^2^ than the other models.

### Kinetic studies

Studying adsorption kinetics is essential for comprehending the mechanisms of adsorption, the time needed to reach equilibrium, and the step that governs the rate of the adsorption process. The rate and mechanism adsorption process can be investigated using various kinetic models.

The Pseudo-first-order model focuses on the capacity of the absorbent and is applicable when adsorption happens through diffusion within a boundary layer. The pseudo-second-order model emphasizes the adsorption occurring on the surface, indicating that adsorption chemical. Pseudo-first-order and second -order models are essential in understanding the complexities of adsorption processes^41^.

**Fig. 12:**
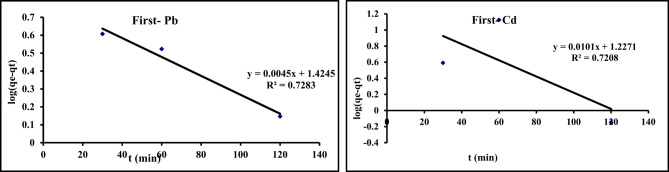
The pseudo-first order kinetic for the adsorption of Pb and Cd on GA/Msc2 nanocomposite.

**Fig. 13:**
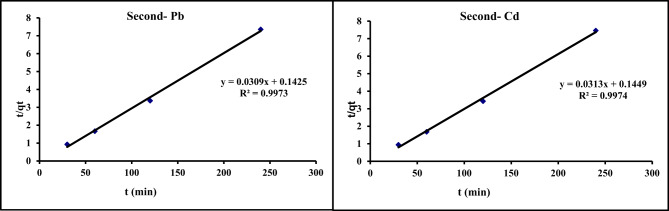
The pseudo-second order kinetic for the adsorption of Pb and Cd on GA/Msc2 nanocomposite.

**Fig. 14:**
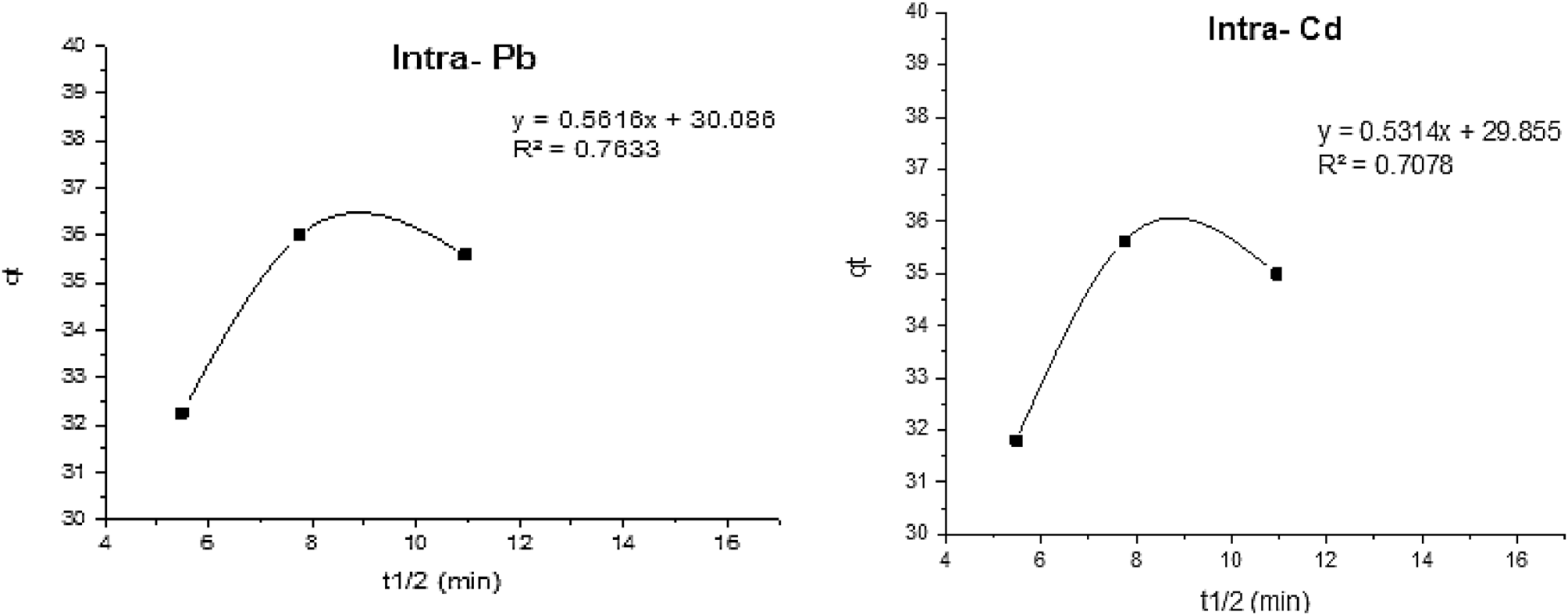
The intra-particle kinetic for the adsorption of Pb and Cd on GA/Msc2 nanocomposite.

**Fig. 15:**
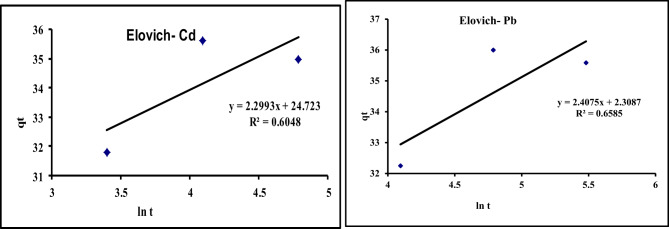
Elovich kinetic model for the adsorption of Cd^2+^ and Pb^2^ on GA/Msc2 nanocomposite.

In this study pseudo-first order, pseudo-second order, intra-particle diffusion, and Elovich models were studied. Kinetic data of Cd^2+^and Pb^2^ adsorption on GA/Msc2 are presented in Table 7, and presented graphically in Figs. 12, 13, 14 and 15.

**Table 7:** Calculated kinetic parameters for the adsorption of Cd^2+^and Pb^2^ on GA/Msc2 nanocomposite.

Parameters	Pb^2+^	Cd^2+^
Pseudo first-order		
K_1_ (min)^−1^	0.01	0.02
q_e_ (mg/g)	4.1	3.4
R^2^	0.73	0.72
Pseudo second-order		
K_2_ (g/mg min)	6.7*10^− 3^	6.7*10^− 3^
q_e_(mg/g) (calculated)	32.3	32.1
q_e_(mg/g) (experimental)	35.6	35
R^2^	0.99	0.997
Elovich model		
α (mg/min)	6.2	6.5
β (g/mg)	0.42	0.44
R^2^	0.66	0.61
Intra particle diffusion model		
K_i_	0.56	0.53
C (mg g^− 1^)	30.1	29.9
R^2^	0.763	0.708

#### Pseudo–first order

From Eq. 7, the constants were obtained from the slope and the intercept of the linear relevance ln(qe − qt) vs. and time (t) (Fig. 12), and tablet in Table 7.

Comparing the R^2^ values of Pb and Cd( 73,74 respectively), it seem nearly equals . Also, rate constant K_1_ of Pb and Cd were 0.01 and 0.02(min)^−1^, respectively. This indicating its superior suitability in explaining the kinetics of Pb and Cd adsorption onto GA/Msc2 nanocomposite.

#### Pseudo–second order

From Eq. (8), by plotting t/qt vs. time (t) (Fig. 13) the straight-line and the parameters R^2^, K_2_ (g/mg min), q_e_ experimental and q_e_ calculated (mg/g) were obtained and cited in Table 7. The higher correlation coefficient values (R^2^) for Cd and Pb than those of Pseudo–first order indicated a best adsorption fitted to pseudo-second order model. Furthermore, the qe calculated values are in close proximity to the qe experimental values, indicating that the overall metal adsorption rate is governed by pseudo-second order, with chemisorption being the rate-limiting factor^42^.

#### Intra-particle diffusion model

From Eq. (9), C and K_i_ obtained from intercept and the slop of the linear plot of q_t_ vs. t^1/2^ (Fig. 14; Table 7). C value is the thickness of boundary layer. This model is not fitting well for adsorption process as the value of R^2^ is low.

The binding constant values (K_i_) indicate that Pb (K_i_ 0.56) has a stronger binding affinity compared to Cd (K_i_ 0.53), which can be attributed to its larger hydrated ionic radius^40^.

#### Elovich model

From Eq. (10), q_e_ vs. ln (t) was plotted (Fig. 15) and α and β are calculated from the plots. The resulted parameters α, β and correlation coefficient (R^2^) are cited in Table 7. Elovich model exhibited low R^2^ values for Pb and Cd (0.66, 0.61, respectively), revealing the unacceptable fitting of this model.

In conclusion of adsorption kinetic models, from Table 7 it indicates that from all four kinetic models applied for adsorption of Cd^2+^ and Pb^2+^ on GA/Msc2 nanocomposite, the pseudo second order kinetic model is only one that fitted well this adsorption. Pseudo-second order kinetics model suggested that the reaction on the adsorbent surface is the rate step of adsorption^43^.

### Thermodynamics of adsorption

**Fig. 16:**
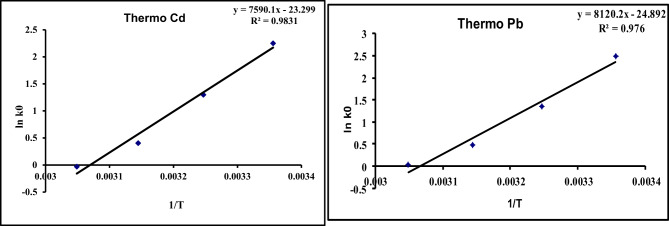
Thermodynamic model for the adsorption of Pb and Cd on GA/Msc2 nanocomposite.

From the results of thermodynamics of adsorption (Table 8, Fig. 16) the values of free energies (ΔG°) for Cd^2+^ and Pb^2+^ at all temperatures were negative, falling within the range of 0 to − 5.8 kJmol^− 1^. This suggests that the mechanism for the adsorption of Cd^2+^ and Pb^2+^ on the GA/Msc2 nanocomposite is physical adsorption.

**Table 8:** Thermo dynamical parameters for the adsorption of Cd^2+^ and Pb^2+^. on GA/Msc2 nanocomposite.

Parameters		Cd	Pb
ΔH^o^, kJ/mol		-13.1	-17.5
ΔS^o^, kJ/ mol K		-0.193	-0.207
ΔG^o^, kJ/mol	298 k	-5.6	-5.8
	308 k	-3.66	-3.7
	318 k	-1.73	-1.7
	328k	-0. 2	-0.4
R^2^		0.983	0.976

The negative ΔH° values indicate that the adsorption process is exothermic, while the lower ΔH0 values suggest that the adsorption of Pb and Cd onto the GA/Msc2 nanocomposite occurs through physical adsorption or physisorptionΔS° values are negative suggested an increase in disorder at the solid-solution interface, where Cd^2+^ and Pb^2+^ were taken up at the active sites of the adsorbent surface.

### The effectiveness of the adsorption by various materials^–^ of the metals under investigation in this study is shown in Table 9

**Table 9:** comparative parameters for the adsorption of Cd, and Pb on different adsorbents.

Adsorbent	Optimum conditions	Adsorption capacity qmax /removal efficiency (%)		References
		Pb²⁺	Cd²⁺	
GG/KPS/AA/EDTA		–	99 mg g^− 1^	^49^
Alginate/gum Arabic composite	pH 6.0, 25 ◦C, 2.5 g L^− 1^ of adsorbent dosage)	1.435(mmol g-^1^)	1.180 (mmol g-^1^)	^3^
Guar gum/bentonite	pH 5.1, contact time 240 min, 300 ppm metal concentration	187.084 mgg^− 1^		^52^
Semi IPN (GG/XG/PAA)	0.05 g of the adsorbent concentration 300 ppm, 27 °C for 24 h.	93%		^46^
Gum Arabic-Magnetite Nanocomposite	0.3 g/50 mL, pH = 6.00, and contact time of 30 min, 50 ppm	50.5 mgg^− 1^		^47^
Polypyrrolebased/Activated carbon AC	pH 5.5, contact time 120 min, concentration 100 mg/l, adsorbent dose 5 g	50 mgg^− 1^		^45^
Polyacrylonitrile and Arabic Gum	pH 5, contact time 2 min, concentration 150 mg/l, adsorbent dose 2 g	1017 mgg^− 1^		^48^
copper terephthalic acid metal-organic framework/gum Arabic/carrageenan composite beads	20 °C, pH 5, 60 min shaking time, 3.0 g/L adsorbent dosage	374.7 mgg^− 1^		^50^
Muscovite based polyaniline nanocomposite	metal concentration 75 ppm, pH 6 and 7 for Pb^2+^ and Cd^2+^, adsorbent dose 0.1 g, 25 °C solution temperature, and 60 min contact time	72.6% 63.1 mgg^− 1^	75.6% 61 mgg^− 1^	^53^
Natural Gum Nanomuscovite Nanocomposite	pH 7, 0.1 g adsorbent dosage, 75 ppm metal concentration, 25 °C solution temperature, and 60 min contact time.	38.01 mgg^1^,96%,	45.9 mgg^− 1^, 95%	This study

## Reaction mechanisms of the interaction of metals in the nanocomposite

Arabic gum/muscovite nanocomposite remove heavy metals primarily through surface adsorption, relying on the synergistic effect between the high surface area of the nanomuscovite and the functional groups of the natural gum (e.g., carboxyl, hydroxyl).

Key mechanisms include electrostatic attraction, cation exchange, complexation, and hydrogen bonding as follows^53^.

*1-Electrostatic Attraction* The negatively charged surface of nanomuscovite (due to isomorphous substitution, such as replacing ) attracts positively charged heavy metal ions.

*2- Cation Exchange* The interlayer spaces of nanomuscovite hold exchangeable cations that are released as heavy metal ions are adsorbed.

*3- Surface Complexation* Functional groups in natural gums (e.g., carboxyl, hydroxyl) form chemical complexes with heavy metals, often acting as binding sites.

*4- Ion Exchange/Chemisorption* The gum-based nanocomposites, especially after grafting or functionalization, enhance surface sites for chemical binding.

*5- Coagulo-adsorption* The composite works by aggregating suspended metals and adsorbing them onto the large surface area of the Arabic gum/muscovite hybrid.

### Synergistic behavior

The nanocomposite combines the high cation exchange capacity of the nanomuscovit with the functional flexibility of gum, leading to high-efficiency removal of Pb and Cd. Factors like strongly affect the dissociation of functional groups and surface charge.

## Conclusion

This study shows modified synthesis of GA/Msc2 nanocomposite and its application for of Pb and Cd removal from solution. Several analyses FTIR, SEM, BET, and XRD were used for characterizing the adsorbent structures. In this study high removal efficiency of Cd^2+^ and Pb^2+^ on GA/Msc2 nanocomposite (95% and 96%, respectively) was obtained at optimal adsorption conditions pH 7, adsorbent dose 0.1 g, metal concentration 75 ppm, contact time 60 min, and solution temperature 25 °C. Langmuir isotherm model and pseudo-second-order model provided good fits for the adsorption of these metals. The thermodynamic studies show exothermic and spontaneous nature of adsorption .

## Data Availability

The authors declare that the data supporting the findings of this study are available within the paper and its supplementary information file. Should any raw data files be needed in another format they are available from the corresponding author upon reasonable request.
